# Defining Physician–Nurse Efforts toward Collaboration as Perceived by Medical Students

**DOI:** 10.3390/healthcare11131919

**Published:** 2023-07-03

**Authors:** Hanan H. Dahlawi, May M. Al obaidellah, Najwa Abdur Rashid, Amal A. Alotaibi, Eman M. Al-Mussaed, Mary Mae M. Cheung, Sameera Abuaish, Mary Anne Wong Cordero

**Affiliations:** 1 Department of Basic Sciences, College of Medicine, Princess Nourah Bint Abdulrahman University, Riyadh 11671, Saudi Arabia; hhdahlawi@pnu.edu.sa (H.H.D.); mmalobaidellah@pnu.edu.sa (M.M.A.o.); nabdurashid@pnu.edu.sa (N.A.R.); amaalotaibi@pnu.edu.sa (A.A.A.); emalmussaed@pnu.edu.sa (E.M.A.-M.); syabuaish@pnu.edu.sa (S.A.); 2 College of Arts and Sciences, Notre Dame of Dadiangas University, General Santos City 9500, Philippines; mmcheung@nddu.edu.ph

**Keywords:** physician–nurse collaboration, attitudes toward collaboration, Jefferson scale of attitudes, interprofessional education, interprofessional collaboration, teamwork

## Abstract

Collaboration between physicians and nurses is essential to healthcare delivery and is associated with high-quality patient care, greater patient satisfaction, and better health outcomes. Hence, it is imperative that doctors and nurses have a particular set of interprofessional collaboration skills. This descriptive cross-sectional study assessed how medical students in the pre-clinical and clinical years perceived attitudes toward collaboration between physicians and nurses in a hospital setting. The Jefferson Scale of Attitude toward Physician–nurse Collaboration (JSAPNC) was reverse-translated into Arabic for the current study. The results showed a total JSAPNC mean score of 46.55, lower than other medical students in other universities. In general, the results of the study showed no significant difference in the total JSAPNC score among medical students when analyzed according to age, clinical exposure, and year level, except in the two factors of JSAPNC: shared education and teamwork (*p* = 0.038) and caring as opposed to curing (*p* = 0.043). The findings of this study suggest the necessity of integrating interprofessional education (IPE) across the medical school curriculum because, as future physicians, medical students would be well equipped to treat their patients in partnership with their nursing colleagues.

## 1. Introduction

Interprofessional collaboration in healthcare is a holistic process encompassing teamwork, communication, and cooperation based on shared power and authority [1]. It is a crucial component of healthcare care and is linked to patients experiencing better health outcomes [2]. In order to deliver the most excellent level of healthcare across settings, several healthcare professionals from varied backgrounds should collaborate with patients, their families, and the community to provide comprehensive services [3]. Even more so, doctors and nurses need specific interpersonal and communication skills and training in interdisciplinary collaboration. These skills and training will enable them to work cooperatively, share responsibilities, solve problems, and make decisions to carry out actions focused on the care of patients [4]. Mutual respect, trust, and efficient communication are essential for a successful collaborative process [5]. The importance of a collaborative approach in professional practice should be highlighted because doctors and nurses collaborate on patient care and have complementary roles [6]. Healthcare team members must be aware of the other professions with which they work. Effective collaboration in healthcare requires deliberate information sharing and shared accountability for patient care [7].

The importance of physician–nurse teamwork and collaboration in producing exceptional clinical outcomes and high-quality patient care has been supported by several studies [8,9]. In a collaborative relationship, the doctor and nurse share duties, work out issues and decide how to create and carry out patient care plans. Both parties must have equal decision-making authority, accountability, and power to manage patient care effectively. There must be evidence of the parties’ mutual regard, trust, and open communication [5]. Members must appreciate one another’s opinions and knowledge in order to win each other’s respect [1]. Additional elements affecting physician–nurse collaboration is job prioritization, comprehension of professional responsibility, respect, and equal power [10]. Providing high-quality patient care that improves outcomes requires effective cooperation and positive connections [11,12,13]. As a result, there are fewer deaths, as it ensures patient security, satisfaction, and speedy recovery [14,15].

Additionally, positive doctor–nurse interactions improved drug utilization and reduced behavioral disturbances in several nursing home residents [16]. It has been demonstrated that ineffective physician–nurse collaboration irritates medical professionals at work and lowers the quality of patient treatment [10]. The views and attitudes of doctors and nurses should be similar regarding teamwork. However, numerous studies demonstrate differences in perspectives and attitudes concerning doctor–nurse collaboration. They disagree on what constitutes an effective working partnership [17]. Nurses in countries where the complementary model of professional responsibilities is more prevalent, such as the United States, were more favorable toward physician–nurse collaboration than nurses in countries where the hierarchical model is more prevalent, such as Italy and Mexico [18]. In Japan, there are still dominant dependent relationships between doctors and nurses regarding difficulties involving nurse–physician collaboration [19]. Despite cultural differences, nurses prioritize doctor–nurse collaboration more than doctors [18]. Nurses are more enthusiastic about working together than doctors [20,21], whereas physicians view collaboration as less critical [22].

In the past, nursing–physician relationships were characterized by physician authority and nurse compliance, with doctors being represented as paternal and directive. Nurses were only supposed to concentrate on patient care and follow the doctors’ orders [17,23]. For instance, the communication hierarchy between doctors and nurses in Middle Eastern countries shows that nurses act as doctors’ assistants [24]. However, the need for collaboration between nurses and doctors is currently emphasized in nursing schools [25]. Due to the complexity of patient care in today’s culture, nurse–physician collaboration necessitates that nurses and doctors coordinate patient care to ensure quality and safety. Supporting nurses’ independence and developing their practical nursing skills are essential for developing professional knowledge and autonomous behavior.

Therefore, it is imperative to pursue an education that emphasizes student specialization and collaboration [19]. Interprofessional education (IPE) should be incorporated into the curricula of medical and nursing schools to promote an understanding of the complementary roles of doctors and nurses and to facilitate the growth of an interdependent relationship between them [26]. Medical and nursing students must take IPE courses [27], and hospital management should offer continuing IPE and cooperation opportunities for all interdisciplinary team members [17]. Interprofessional collaboration and teamwork must be practiced across the curriculum and integrated into most instruction to have a more evident impact. As cooperation significantly impacts medical students’ perception, they must be regularly reminded of it when practicing in the wards and healthcare facilities [9]. Additionally, their views regarding IPE significantly influence student attitudes during interprofessional practice. Therefore, medical students need interprofessional training from the first year of study to better grasp professional roles and duties, effective communication, and teamwork [28].

As mentioned in the preceding paragraphs, effective health care requires interdisciplinary collaboration and teamwork; therefore, looking into medical students’ perceptions of physician–nurse collaboration is crucial. Teaching medical students to collaborate effectively within a team may help to assure the delivery of high-quality healthcare, as doctors and nurses play a vital role in patient care. It is necessary to investigate medical students’ perspectives on collaboration in Middle Eastern countries because interprofessional partnerships between nursing and medicine often follow a hierarchical structure. Doctors often predominate in patient care decisions, while nurses have less discretion. Hence, this study was designed to assess the attitudes of medical students toward collaboration using the Jefferson Scale of Attitudes toward Physician–Nurse Collaboration (JSAPNC). Specifically, measuring attitudes considered the four components of JSAPNC: shared education and teamwork; caring as opposed to curing; nurses’ autonomy; and physician authority. Factors influencing medical students’ attitudes towards collaboration, such as age, year level, and clinical exposure, were also investigated.

As part of its ongoing pursuit of quality medical education, the College of Medicine at Princess Nourah bint University (PNU) regularly conducts curriculum assessments of its Bachelor of Medicine and Surgery (MBBS) program. The current study’s results may be utilized further to enhance the existing curricular content relevant to interprofessional collaboration. Moreover, the findings of this study may be used as a benchmark for developing study curricula and IPE training sessions tailored specifically to the requirements of healthcare professionals. Integrating efficient IPE allows the students to develop the interprofessional skills needed for collaboration across disciplines.

## 2. Materials and Methods

### 2.1. Study Design

This descriptive cross-sectional study examined how medical students in the pre-clinical and clinical years perceived teamwork and collaboration between physicians and nurses in a hospital setting.

### 2.2. Study Participants and Setting

In total, 256 completed questionnaires were considered for data analysis. All study participants were female, aged 23.11 ± 1.03, as Princess Nourah bint Abdulrahman University (PNU) is a women’s university. The study included 63 first-year, 58 second-year, 47 third-year, 46 fourth-year, and 42 fifth-year medical students from PNU, College of Medicine.

Since its commencement in 2012, the Bachelor of Medicine and Surgery program (MBBS) of the College of Medicine at PNU in Saudi Arabia has followed a unique problem-based hybrid curriculum. The program starts with a two-year pre-clinical Block System. First-year courses include the Foundation Block, Musculoskeletal, Cardiovascular, Respiratory, and Renal courses. In the second year, students take the Central Nervous System, Endocrine, Gastrointestinal and Hematology, and Reproduction blocks. Anatomy, physiology, biochemistry, genetics, pathology, microbiology, pharmacology, learning skills, and medical professionalism are the courses offered in each block following the weekly themes. There are lectures, tutorials, and practical or laboratory sessions for several courses within each block. Self-directed learning (SDL), problem-based learning (PBL), and clinical skill sessions, such as intramuscular injection (IM) and cardiopulmonary resuscitation (CPR), are all integrated.

The students enroll in clinical courses from the third to the fifth year of the MBBS program. During this time, they take courses such as internal medicine, community medicine, obstetrics and gynaecology, primary health care, general surgery, paediatrics, and other specialities. Students are exposed to various clinical settings in various government and private hospitals throughout the kingdom during the clinical years. Table 1, below, shows the different learning skills and medical professionalism lectures offered during the MBBS program.

### 2.3. Instrument

Medical students’ perceptions of doctors’ and nurses’ attitudes toward collaboration were assessed using the Jefferson Scale of Attitudes toward Physician–Nurse Collaboration (JSAPNC). Initially developed [29] to assess attitudes toward nurses and nursing services, this questionnaire was later amended [30] to examine attitudes regarding physician–nurse relationships. Following rigorous psychometric research [31], additional questionnaire adjustments were made, keeping 15 of the original 20 items. Interprofessional collaboration is viewed as a cooperative endeavor with shared authority and responsibility, open communication, and shared decision-making. This is the JSAPNC’s fundamental principle. The attitudes of doctors and nurses toward one another may change due to professional training in a collaborative setting [30]. In the JSAPNC instrument, seven items—1, 3, 6, 9, 12, 14, and 15—were classified as “shared education and teamwork,” three—2, 4, and 7—were classified as “caring rather than curing,” five—5, 11, and 13—were classified as “nurse’s autonomy,” and items 8, and 10 were classified as “physician’s authority.”

Thirteen (13) of the fifteen (15) items were considered to have favorable attitudes. The responses had the following direct coding: 4 = strongly agree, 3 = agree, 2 = disagree, and 1 = strongly disagree. It was established that two of the measures (items 8 and 10) reflected unfavorable attitudes toward nurse–physician teamwork. The responses to these questions were scored in the opposite (1 = strongly agree, 2 = agree, 3 = disagree, and 4 = disagree). The sum of all the item scores is the overall score. The higher the score, the more supportive the physicians and nurses are of working together. A higher factor score on shared education and teamwork indicates an excellent attitude toward multidisciplinary education and interprofessional partnerships. A higher factor score on caring than curing indicates a more favorable opinion of nurses’ contributions to the psychological and educational aspects of patient care. A higher factor score on the nurses’ autonomy indicates greater agreement with nurses’ participation in choices about patient care and policies. Higher physician dominance factor scores indicate a rejection of a completely dominant position for doctors in patient care.

The JSAPNC was reverse-translated into Arabic for the current study’s purposes to show semantic comparability and increase the validity of the questionnaire. Two independent, bilingual individuals translated the questionnaire into Arabic, and the researchers then assessed the quality of the translation. Discussions with the translators helped to find and fix translation errors. Two additional competent translators who had not read the original questionnaire completed the backward translation. The Arabic version of the instrument had a pilot test with the participation of fifteen (15) medical students from various year levels. Minor comments from the pilot research served as the basis for a slight modification, such as rewording the two Arabic questions and making the instructions more explicit. The data gathered from the pilot test were not included in the final results.

Internal consistency was examined using the coefficient alpha or Cronbach’s alpha to see if the translated Arabic version of the JSAPNC instrument could be used among the medical students of the current study.

### 2.4. Data Collection and Analysis

The medical students were given access to an Arabic-language online version of the JSAPNC instrument from 7 March to 28 April, the conclusion of the second semester of the academic year 2021–2022. Each student’s permission to participate in the study was requested before the questionnaire was sent. The participants were informed about the format of the JSAPNC instruments and the voluntary nature of their participation in the study. The questions were completed online by the respondents, who then submitted them. However, the data analysis did not include incomplete forms. A respondent must answer at least 12 or 80% of the questionnaire’s 15 items for it to be deemed complete and considered throughout the data analysis.

The data were analyzed using descriptive statistics such as frequencies, means, and standard deviation. The difference of means in different year levels of medical students was computed, then the total and factor scores on the JSAPNC Scale were identified and evaluated using Analysis of Variance (ANOVA), with *p*< 0.05 considered significant. The assumption of the normality of the data was considered for validation of the data gathered in the study. An assessment of the normality assumption of the distribution of the JSAPNC scores was conducted prior to subjecting the data to an analysis of variance using the Shapiro-Wilk and Kolmogorov-Smirnov tests. Data analysis was conducted using the SPSS version 20 (Statistical Package for Social Sciences 20).

## 3. Results

### 3.1. Reliability of the Arabic Version of the JSAPNC Instrument and Distribution Normality of JSAPNC Score

The Arabic version of the JSAPNC instrument used in this study yielded Cronbach’s alpha coefficients ranging between 0.76 and 0.89, demonstrating an acceptable internal consistency.

The detection of outliers in the data set was conducted by examining and analyzing the histogram and box plot. Far-out outliers identified with a “star” were automatically removed. The normality of our data was assessed using the Shapiro–Wilk, Kolmogorov-Smirnov, and Levene’s tests, shown in Table 2. The *p*-values for these tests are greater than 0.05, assuming the data are normally distributed.

### 3.2. The Attitude of Medical Students toward Physician–Nurse Collaboration

**Figure 1:**
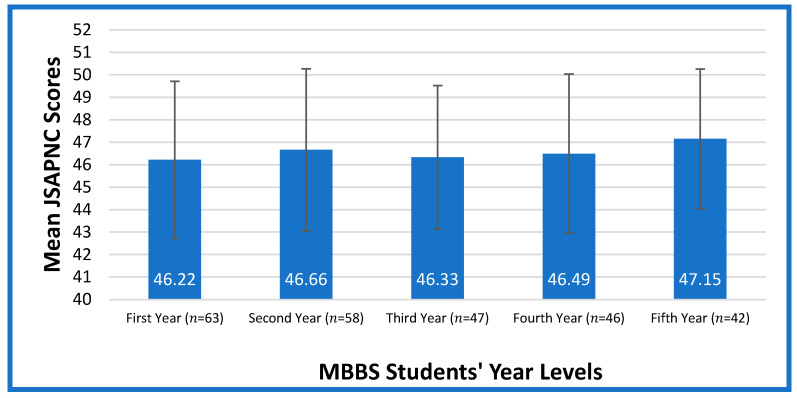
Medical Student’s Attitude Toward Physician-nurse Collaboration.

Based on the JSAPNC instruments’ scoring algorithm, the higher the score, the more favorable the respondents’ attitudes were toward physician–nurse collaboration. Figure 1 displays the mean ratings of the medical students’ attitudes toward collaboration between doctors and nurses in a hospital setting. According to the results, first-year medical students have the lowest JSAPNC mean score (46.22), while fifth-year medical students have the highest (47.15). The findings of this study revealed that fifth-year medical students had a more positive attitude toward collaboration compared to the lower years, except for the second year, who scored higher than the third- and fourth-year students.

### 3.3. Difference in the Attitude of the Medical Students towards Physician–Nurse Collaboration When Analyzed According to Age, Year Level, and Clinical Exposure

The study’s findings showed no significant difference in the attitudes toward collaboration among medical students when analyzed according to age (*p =* 0.164) and clinical exposure (*p =* 0.197). However, a significant difference was observed in the two factors of the JSAPNC, shared education and teamwork (*p =* 0.038) and caring as opposed to curing (*p* = 0.043), but not in nurse autonomy and physician authority. In general, the JSAPNC total score showed no significant difference in the perceived attitude of medical students toward physician–nurse collaboration when compared to the year level. The analysis of the medical students’ attitude to physician–nurse collaboration in the four components of the JSAPNC is summarized in Table 3.

When the results are broken down according to year level, they reveal a considerable difference between the two JSAPNC factors: shared education and teamwork and caring as opposed to curing. To further investigate any significant differences among the medical students according to their year level, a post-hoc test (Tukey test) was carried out. An alpha level of 0.05 was applied for the entire set of tests. The outcomes of the post-hoc test is shown in Table 4. Under shared education and teamwork, a significant difference (*p* = 0.045) in the attitude towards physician–nurse collaboration was only observed between the first year and fifth-year students. However, in caring as opposed to curing, significant differences among the groups were observed, except between the first and second years, first and third years, and fourth and fifth years.

The effect size was similarly calculated in the two JSAPNC factors, shared education and teamwork and caring as opposed to curing. The effect size for shared education and teamwork, calculated as eta squared (η^2^), was 0.037, indicating a small effect. For caring as opposed to curing, η^2^ was 0.108, indicating a moderate effect.

## 4. Discussion

This study used the JSAPNC scale to evaluate medical students’ perceptions of physician–nurse collaboration in hospital practice. The following four factors were considered: shared learning and teamwork, caring rather than curing, nurse autonomy, and physician authority. The effects of age, year level, and clinical exposure on the medical students’ views toward collaboration were also examined. The study results may serve as the basis for creating curricula and IPE training sessions tailored to healthcare professionals’ needs.

The results showed a total JSAPNC mean score of 46.55, which is close to the total JSAPNC of 47.26 from the medical students at Chongqing Medical University in China [26], and Mexican medical students, with a JSAPNC score of 47.0 [32], where the hierarchical model of professional responsibilities may still be common. However, this result is lower when compared to the total JSAPNC mean score of 48.0 among medical students reported in another study [33], where the complementary model is more practiced. These results may suggest a hierarchical relationship between doctors and nurses characterized by the doctors’ dominance over nurses, who are considered doctors’ assistants instead of collaborators for holistic patient care [6,24,34].

According to a recent study, without targeted training programs, the sociocultural environment greatly influences how people develop their collaboration skills [4]. In regions such as the Middle East, where physicians predominate in decisions regarding patient care and nurses have little autonomy [6], this influence is a significant cause for concern. Moreover, this influence can be strengthened in academic settings where medical training mostly focuses on technical and clinical skills over communication and social work aptitudes [4,32]. Hence, it is a significant educational concern to determine how medical students develop their attitudes toward interprofessional collaboration with nurses, which is influenced by the extent of their exposure to a hierarchical setting [35]. In this situation, physicians have autonomy concerning patient care. Therefore, medical and nurse schools must incorporate or enhance IPE in their curricula to augment the understanding of the complementary roles of physicians and nurses [26]. This will foster the formation of interdependent relationships between doctors and nurses. As reflected in Table 1, in our MBBS program, lectures related to communication skills and interprofessional relationships are given during the first and second years. More targeted training and sessions may be needed for collaborative skills development and amalgamating communication, teamwork, and collaboration skills into our problem-based learning (PBL). Additionally, collaboration and teamwork must be practiced across the medical curriculum and integrated into most instruction to have a more visible impact [36].

The study’s findings showed no significant difference in the attitude of medical students toward physician–nurse collaboration when analyzed according to age. The age of the study participants ranged between 18 and 26, which may be narrow enough to warrant a significant difference. However, when the two JSAPNC components were examined according to year level, a significant difference in the students’ views about collaboration was found in shared education and teamwork (*p* = 0.038) and caring as opposed to curing (*p* = 0.043) but was not significant in the overall JSAPNC score. The difference in attitude across the year levels may be due to many factors, including but not limited to a lack of theoretical and IPE skill instruction for students, a knowledge gap, and an amalgamation of the student’s individual experiences and attitudes [4]. Additionally, students’ perspectives on interprofessional cooperation, interdisciplinary education, and nurses’ contributions to patient care’s psychological and educational aspects may vary.

There was no significant difference in the medical students’ attitudes about physician–nurse collaboration when the results were broken down according to the clinical exposure and year level. These outcomes corroborate those of a different study that used the JSAPNC scale to assess variations in medical students’ attitudes toward collaboration between physicians and nurses at two medical schools. According to the reports, final-year students at both colleges did not show a more positive attitude towards collaboration than first-year students [36]. Additionally, medical students exposed to an interprofessional curriculum for 12 weeks spread over 3 phases in 5 years showed the same attitude towards collaboration as those without exposure [36]. In a different South Korean study, the overall JSAPNC score did not change significantly among medical students but increased significantly among nursing students after finishing simulation-based IPE [37]. In contrast, another study found that Lithuanian medical students had a greater knowledge of nurses’ functions after completing six months of interprofessional training alongside nurses in the hospital. However, in a foreign medical program of the same medical college that did not offer interprofessional training, students highlighted that nurses performed more efficiently alone than in a team, using primarily technical abilities, and communicated inadequately with patients [28]. These differences in the success of IPE integration may be due to a language barrier and cultural differences [4,29].

The current study’s findings point to the necessity of integrating interprofessional education (IPE) into our MBBS curriculum. IPE is the participation of two or more healthcare professionals in a collaborative learning environment to improve health [3]. It is an essential strategy for preparing students for careers in the health sector, where teamwork and collaboration are crucial skills. Several international health organizations have advocated for IPE to encourage interprofessional teamwork, increase the standard of patient care, and better health outcomes [38]. IPE can be broadly categorized as being classroom-, simulated-, and practice-based [39], preparing students for interprofessional collaboration (IPC). IPC brings together multiple individuals from diverse backgrounds, such as doctors and nurses, to collaborate to deliver the best possible healthcare [3,39]. Efficient IPC supports an environment for teamwork that upholds safe and better-quality patient-centered care [40]. With a comprehensive integration of IPE into the medical curriculum, it is envisioned that future graduates will exemplify collaborative work attitudes and behavior. In fact, communication and collaboration are one of the six required competencies in the Saudi Medical Education Directives Framework (SaudiMed Framework). The framework serves as a national benchmark for Saudi medical graduates and is designed to ensure that they have acquired the skills needed to succeed as doctors [41]. One of the six themes in the SaudiMed Framework is communication and collaboration competency. Under this is program learning outcome 11 (PLO 11), which specifies the practice of teamwork and interprofessional collaboration among medical graduates. Included in the PLO 11 is the achievement of the four course learning outcomes (CLOs), as follows: (1) to collaborate and identify the roles of various healthcare professionals involved in patient care and to collaborate with them; (2) to collaborate and identify the roles of various healthcare professionals involved in patient care and collaborate with them; (3) to recognize the benefits of teamwork and emphasize their importance; and (4) to demonstrate the ability to prevent and resolve interprofessional team conflicts.

It is worth noting that the students’ lower JSAPNC total scores in this study, compared with other studies, cannot be generalized as a reflection of a “poor” or “negative” attitude toward physician–nurse collaboration. The interplay of other factors, such as culture, psychological aspects, and environment, is still to be explored. Hence, longitudinal and qualitative studies such as interviews and focus group discussions may be conducted to further elucidate the attitude of medical students towards physician–nurse collaboration. Nevertheless, it is imperative to note some recommendations in Table 5 vis-à-vis the integration of IPE in the medical curriculum to develop collaborative skills among students.

## 5. Conclusions

The attitudes of the medical students in this study toward physician–nurse collaboration across year levels are lower than other students from other medical colleges. These results imply that students need to be more efficiently provided with the interprofessional education and training that they need to become future collaborative team members in the healthcare industry. Despite the ample integration of teamwork and collaboration content in our MBBS curriculum, there is a need to revisit the curricular content in different courses across all year levels to better implement and assess IPE and incorporate it into teaching and training. It is imperative that the College of Medicine and Nursing requires interprofessional educational courses between medical and nursing students. In addition, hospital administrators need to offer ongoing IPE and collaboration experiences for all interdisciplinary team members because effective health care depends on multidisciplinary collaboration and teamwork.

## Figures and Tables

**Table 1:** Lectures and instructional activities related to collaboration and teamwork in MBBS Program.

Lectures and Instructional Activities	Number of Hours
Learning Skills:	
Group dynamics	1
Become a PBL Student	1
Communication skills	1
Group Activity (presentation)	3
Medical Professionalism:	1
The concept of communication skills	1
Dealing with different types of patients	1
Dealing with patients in difficult situations	1
Leadership in healthcare	1
Interprofessional relationship	1
Concepts of community service and social accountability	1
Group Activity (Brochure & Poster Presentation)	3

**Table 2:** Tests for the analysis of normality.

JSAPNC Factors	Kolmogorov-Smirnov		Shapiro-Wilk		Levene	
	Statistics	*p*-Value	Statistics	*p*-Value	Statistics	*p*-Value
Shared Education and Teamwork	0.132	0.079	0.973	0.184	0.542	0.105
Caring as Opposed to Curing	0.102	0.203	0.976	0.262	0.380	0.823
Nurse Autonomy	0.135	0.074	0.928	0.143	0.493	0.214
Physician Authority	0.104	0.089	0.970	0.121	0.392	0.802

**Table 3:** Test of difference in the attitude of medical students to physician–nurse collaboration when analyzed according to year level.

JSAPNC Factors	Year Level	Mean ± SD	*F*-Value	*p*-Value
Shared Education and Teamwork	First Year	22.20 ± 2.25	4.536	0.038 *
	Second Year	22.77 ± 2.38		
	Third Year	22.91 ± 2.29		
	Fourth Year	22.33 ± 2.41		
	Fifth Year	23.37 ± 2.05		
Caring as Opposed to Curing	First Year	8.86 ± 1.38	4.413	0.043 *
	Second Year	8.98 ± 1.47		
	Third Year	8.77 ± 1.35		
	Fourth Year	9.78 ± 1.51		
	Fifth Year	9.90 ± 1.40		
Nurse Autonomy	First Year	10.63 ± 1.25	1.993	0.469
	Second Year	10.63 ± 1.36		
	Third Year	10.58 ± 1.38		
	Fourth Year	10.24 ± 1.41		
	Fifth Year	10.07 ± 1.31		
Physician Authority	First Year	4.53 ± 1.12	1.828	0.146
	Second Year	4.28 ± 1.27		
	Third Year	4.07 ± 1.16		
	Fourth Year	4.16 ± 1.21		
	Fifth Year	3.81± 1.01		
Total Score	First Year	46.22 ± 3.49	1.396	0.153
	Second Year	46.66 ± 3.61		
	Third Year	46.33 ± 3.19		
	Fourth Year	46.49 ± 3.54		
	Fifth Year	47.15 ± 3.11		
Total JSAPNC Mean Score		46.55		

* Significant at *p* ≤ 0.05.

**Table 4:** Multiple comparisons of medicals students’ attitudes to physician–nurse collaboration.

Shared Education and Teamwork		Mean Difference	Standard Error	*p* Value
First Year	Second Year	0.589	0.338	0.552
	Third Year	0.745	0.411	0.368
	Fourth Year	0.172	0.413	0.994
	Fifth Year	1.18	0.424	0.045 *
Second Year	Third Year	0.156	0.418	0.996
	Fourth Year	0.416	0.421	0.860
	Fifth Year	0.594	0.432	0.644
Third Year	Fourth Year	0.572	0.442	0.695
	Fifth Year	0.438	0.452	0.869
Fourth Year	Fifth Year	1.01	0.455	0.175
Caring as Opposed to Curing				
First Year	Second Year	0.152	0.254	0.975
	Third Year	0.025	0.269	1.00
	Fourth Year	0.969	0.271	0.004 *
	Fifth Year	1.06	0.278	0.001 *
Second Year	Third Year	0.178	0.274	0.967
	Fourth Year	0.817	0.276	0.028 *
	Fifth Year	0.915	0.283	0.012 *
Third Year	Fourth Year	0.995	0.290	0.006 *
	Fifth Year	1.09	0.297	0.003 *
Fourth Year	Fifth Year	0.098	0.292	0.997

* Significant at *p* ≤ 0.05.

**Table 5:** Recommendations to strengthen IPE with a focus on teamwork and collaboration in the MBBS curriculum.

Areas for Consideration	Recommendations
The present MBBS curriculum is enriched with teamwork and collaboration content in different courses from pre-clinical to clinical. However, they are mostly classroom-based, involving uniprofessional student group-based activities except during the Foundation course, where students experience interprofessional student groups.	Despite including teamwork and collaboration content in different courses, it is worth looking into more areas in the instruction where we can further enhance collaborative skills among medical students. To consider the integration of simulated and practice-based IPE into the curriculum as students progress into a higher level of learning and practice utilizing interprofessional student group-based activities. This will help guarantee that students entirely use the interprofessional experience [38].
The current MBBS curriculum reflects the SaudiMed Framework of the PLO 11 and the 4 CLOs under it, as mentioned in the preceding paragraph but needs to be clearly defined and focused in most courses.	To strengthen the application of the SaudiMed Framework in the different curricular activities, putting more emphasis on competencies for collaborative practice, considering interprofessional knowledge, skills, and attitudes [3]
Different teaching-learning activities & strategies in the current curriculum include lectures, PBL, team-based learning, tutorials, group discussions, case-based learning, demonstrations, hospital rotations, bedside teaching, and seminars. Despite these variations, there is a need to look deeper into the effectiveness of these methods vis-a-vis the development of students’ collaborative skills.	Educators must undergo training on developing, delivering, and assessing IPE. It is essential that they comprehend the philosophy, principles, and methods of IPE and adjust their teaching strategies to interact with and direct student learning for different professions [38]. Using flipped classroom method to IPE offers advantages because students receive the same pre-class material and come to class with assumed knowledge. By doing this, more time in the classroom can be devoted to student-centered learning, giving the facilitator more opportunities to encourage the development of students’ knowledge and skills. To integrate more simulations and clinical case studies across year levels because they were highlighted as viable methods to integrate with different healthcare disciplines [42]. To develop simulation experience to allow the medical and nursing students to engage in clinical scenarios representing different patients’ diagnoses, such as in an acute care environment [43]. Participation of medical students in a clinical nursing rotation will provide a reliable method to develop interprofessional skills related to professionalism, collaboration, and communication [44]
Both formative and summative assessments are used in the current curriculum. Examples are; assignments, Continuous assessment in PBL using Rubrics, Mini-CEX, OSCE, and clinical training evaluation.	Despite the diverse assessment methods used, there is a need to focus on evaluating learners’ attainment of clearly defined outcomes for competency in collaborative practice, including interprofessional knowledge, skills, and attitudes at both course and program levels. To provide the learners with accurate and timely feedback on their progress toward achieving IPE outcomes because it is an integral part of the programs for health professional education [45]. Peer feedback exchanges in an interprofessional setting can be effective because the opinions of healthcare professionals from other fields can frequently be insightful and foster self-reflection [46]

## Data Availability

The data used in this study are available and will be provided by the corresponding author at a reasonable request.
